# NT-proBNP as a biomarker for hyperdynamic circulation in decompensated cirrhosis 

**Published:** 2018

**Authors:** Roman Maslennikov, Anastasia Driga, Konstantin Ivashkin, Vladimir Ivashkin

**Affiliations:** *Sechenov First Moscow State Medical University (Sechenov University), Clinic for Internal Diseases, Gastroenterology and Hepatology Pogodinskaya str., 1, bld. 1, 119435, * *Moscow* *, * *Russian Federation*

**Keywords:** Blood circulation, Liver cirrhosis, Biomarkers, Natriuretic peptide, Brain

## Abstract

**Aim:**

To assess NT-proBNP as a biomarker for hyperdynamic circulation in decompensated cirrhosis.

**Background:**

Hyperdynamic circulation is common in decompensated cirrhosis. The previous studies reveal that N-terminal-proBNP (NT-proBNP) is elevated in cirrhosis.

**Methods:**

A prospective study involved 47 patients with decompensated cirrhosis. All of them underwent echocardiography with simultaneous measurement of blood pressure and heart rate. Cardiac output and systemic vascular resistance were calculated. The concentration of NT-proBNP in blood was measured with enzyme-linked immunosorbent assay.

**Results:**

In patients with decompensated cirrhosis, the concentration of NT-proBNP in blood directly correlated with end-diastolic volume (r=0.482; p<0.001), stroke volume (r= 0.566; p<0.001), cardiac output (r=0.556; p<0.001), volume of the left atrium (r=0.292; p=0.047), and inversely correlated with systemic vascular resistance (r=-0.538; p<0.001). There was no significant correlation between NT-proBNP and ejection fraction (p=0.083). Patients with hyperdynamic circulation have higher concentration of NT-proBNP (152÷476 pg/ml vs. 31÷133 pg/ml, p<0.001) regardless of the presence of diastolic dysfunction (p=0.222). According to ROC analysis, the best cut-off values for detection of hyperdynamic circulation in decompensated cirrhosis are considered to be 170.0 pg/ml of blood NT-proBNP, showing sensitivity and specificity of 72.0 and 86.4%, respectively. The positive and negative predictive value are 86.4% and 73.1%, AUC = 0.829 (0.709-0.949).

**Conclusion:**

NT-proBNP may serve as a non-invasive biomarker for hyperdynamic circulation in decompensated cirrhosis.

## Introduction

 Hemodynamic changes in cirrhosis were first described more than 60 years ago (1,2) and include increased cardiac output, increased total blood volume, decreased blood pressure, and decreased systemic vascular resistance. Together, these changes constitute hyperdynamic circulation. Vasodilatation and hyperdynamic circulation are discussed to cause complications of cirrhosis including portal hypertension, hepatorenal and hepatopulmonary syndromes, and hepatic encephalopathy (3). 

Non-selective beta-blockers remain the cornerstone of medical treatment of portal hypertension. However, the effect of non-selective beta-blockers depend on the severity of the hyperdynamic circulation and splanchnic vasodilation (4). No biomarker has been proposed for hyperdynamic circulation in cirrhosis, and hemodynamic parameters, such as cardiac output and systemic vascular resistance, are not usually determined before beta-blockers use. Hepatic venous pressure gradient was supposed for diagnosis of portal hypertension, but the measurement of it, is an expensive and invasive procedure and is not common (4). Thus, beta-blockers are usually used blindfold in cirrhosis. The use of a simple serum biomarker for hyperdynamic circulation may provide differential management with beta-blockers and control for their use, which may increase the effectiveness of therapy and reduce the incidence of side effects.

The endocrine function of the heart was first described in 1981, when the researchers injected the extract from the atria to rats and observed a significant increase in sodium and water excretion with urine (5). Myocardial hormone responsible for this effect was a 28 amino acid polypeptide (6), which was termed the atrial natriuretic peptide. Later, a similar peptide was isolated from the brain tissue and was called the brain natriuretic peptide (BNP) (7).

However, as it was revealed later that despite its name most of the BNP of blood is formed by the myocardium of the heart ventricles (8-9). After a significant increase in blood BNP in the patients with heart failure was described, this peptide began used as a biomarker of this disease (10). The physiological role of BNP is to reduce the volume of circulating blood to prevent cardiac overload. An increase in blood BNP in the patients with decompensated cirrhosis was established in 1992 (11). Most researchers consider BNP as a biomarker of cardiac dysfunction in cirrhosis (12-14).

It was found that the stretching of cardiomyocytes, which is observed in the dilatation of the left ventricle in heart failure, leads to an increase in the release of BNP into the blood (15). Since hyperdynamic circulation is also associated with the dilatation of the left ventricle (16), we assume that BNP in cirrhosis may serve as a biomarker for hyperdynamic circulation. It was shown that BNP is formed from proBNP after its cleavage into BNP and the N-terminal fragment (NT-proBNP) (17). NT-proBNP is more stable, therefore it may have better analytical characteristics. 

The aim of this study is to assess NT-proBNP as a biomarker for hyperdynamic circulation in decompensated cirrhosis. 

## Methods

In this cross-sectional prospective study, 140 consecutive patients with cirrhosis admitted to the Department of Hepatology’s Clinic for Internal Diseases, Gastroenterology and Hepatology (the Clinic), at Sechenov University, Moscow, were screened for inclusion. Study procedures were explained to potential participants and written informed consent was obtained before enrollment. The study had been performed in accordance with the ethical standards laid down in an appropriate version of the Declaration of Helsinki. The study protocol was approved by the Ethical Committee of Sechenov University.

Inclusion criteria were: diagnosis of decompensated cirrhosis verified by histology, clinical, biochemical, and ultrasound findings; age between 18 and 70 years. Severity of liver disease was determined using the Child–Turcotte–Pugh (CTP) scoring system, in which class A was defined as compensated cirrhosis and classes B and C were defined as decompensated cirrhosis.

Exclusion criteria included: cardiac disease, cancer or any other severe disease. 

Of the original 140 patients screened for inclusion, 47 met criteria and were enrolled in the study. The other patients had compensated cirrhosis (n=61), cancer (n=4), other severe disease (n=9), and 19 patients declined to participate.

The concentration of NT-proBNP in blood was measured with enzyme-linked immunosorbent assay.

Echocardiography was performed at rest according to guidelines published by The American Society of Echocardiography (18). End-diastolic volume and end-systolic volume of the left ventricle were determined according to modified Simpson's disk method. Ejection fraction of the left ventricle was calculated as ((End-diastolic volume) - (End-systolic volume))/(End-diastolic volume). Stroke volume was calculated as (Doppler velocity time integral) × (cross-sectional aorta area). Systolic and diastolic blood pressure and heart rate were measured during stroke volume measurement using automatic oscillometric sphygmomanometer (AND, Japan). Mean arterial pressure was calculated as ((Systolic blood pressure) + 2 × (Diastolic blood pressure))/3. Cardiac output was calculated as (Stroke volume) × (Heart rate). Systemic vascular resistance was calculated as (Mean arterial pressure) / (Cardiac output). Diastolic dysfunction Grade I-IV was determined according to recent guideline (19).

Statistical analysis was performed with STATISTICA 10 software (StatSoft Inc., USA) and SPSS (IBM, USA). The differences between continuous variables were assessed with the Mann-Whitney test. Correlations between variables were computed using Spearman's rank correlation. Fisher's exact test was used to assess the difference between categorical variables. Data were represented as mean ± SD, except for NT-proBNP concentration, which was represented as interquartile range. P-value < 0.05 was considered statistically significant. 

## Results

The main parameters of participants are presented in Table 1. Blood NT-proBNP directly correlated with end-diastolic volume (r = 0.482; p <0.001), stroke volume (r = 0.566; p <0.001), cardiac output (r = 0.556; p <0.001), volume of the left atrium (r = 0.292; p = 0.047), and inversely correlates with systemic vascular resistance (r = -0.538; p <0.001). There were no significant correlations between NT-proBNP and ejection fraction (r = 0.226; p = 0.083), heart rate (p=0.087), and mean arterial pressure (p=0.151).

**Table 1:** The main parameters of participants

	Cirrhosis class B CTP (n=27)	Cirrhosis class C CTP (n=20)	p-value
CTP score	7.86±0.86	10.90±1.29	<0.001
Age, year	49.6±11.9	49.4±10.2	0.914
Male/female	12/15	16/4	0.015
Etiology: alcohol	16	14	0.328
viral	4	2	0.489
autoimmune	1	1	0.675
mixed	5	2	0.352
cryptogenic	1	1	0.675
End-diastolic volume, ml	115.1±19.6	126.0+31.4	0.302
End-systolic volume, ml	46.0±8.7	49.5±14.3	0.426
Ejection fraction, %	60.0±4.3	60.5±7.3	0.519
Stroke volume, ml	69.2±15.3	76.5±21.8	0.208
Heart rate, bpm	82.1±10.7	87.5±11.4	0.179
Cardiac output, l/min	5.71±1.45	6.77±2.38	0.168
Systolic blood pressure, mm Hg	111.3±9.8	110.5±9.4	0.739
Diastolic blood pressure, mm Hg	72.4±6.4	70.5±7.6	0.420
Mean arterial pressure, mm Hg	85.4±7.1	83.8±7.7	0.533
Systemic vascular resistance, dyn х sec/cm^5^	1277±352	1125±443	0.126
Left ventricular dilatation (present/absent)	8/19	6/14	0.613
Volume of the left atrium, ml	30.0±8.5	38.3±12.9	0.005
Left atrial dilatation (present/absent)	5/22	10/10	0.024
Hyperdynamic circulation (present/absent)	14/13	11/9	0.533
Е/А	1.08±0.29	1.09±0.21	0.755
Systolic heart dysfunction	1	1	0.675
Diastolic heart dysfunction Grade I	6	2	0.242
Diastolic heart dysfunction Grade II	4	8	0.053
Diastolic heart dysfunction Grade III-IV	0	0	1.000
NT-proBNP, pg/ml	76.3÷311.9	56.8÷298.7	0.739

**Table 2:** Main hemodynamic parameters according to blood NT-proBNP level

	Elevated NT-proBNP (n=26)	Normal NT-proBNP (n=21)	p-value
End-diastolic volume, ml	128.6±26.5	109.2±20.3	*0* *.* *014*
End-systolic volume, ml	49.2±11.8	45.3±10.8	0.369
Ejection fraction, %	61.6±4.2	58.5±6.7	0.131
Stroke volume, ml	79.1±17.5	63.9±14.3	*0.003*
Heart rate, bpm	85.6±11.5	82.9±10.9	0.380
Systolic blood pressure, mm Hg	110.8±9.8	111.2±9.5	0.940
Diastolic blood pressure, mm Hg	71.1±7.1	72.1±6.8	0.653
Mean arterial pressure, mm Hg	84.4±7.3	85.2±7.4	0.692
Volume of the left atrium, ml	36.8±13.7	28.1±4.8	*0* *.* *049*
Left atrial dilatation (present/absent)	14/12	1/20	*0* *.* *006*
Cardiac output, l/min	6.88±2.13	5.27±1.25	*0.* *007*
Hyperdynamic circulation (present/absent)	19/7	6/15	*0* *.* *003*
Systolic heart dysfunction	0	2	0.194
Diastolic heart dysfunction Grade I	3	5	0.235
Diastolic heart dysfunction Grade II	12	0	*<0.* *001*
Systemic vascular resistance, dyn х sec/cm^5^	1083±374	1368±373	*0.014*

Significant differences are marked in italics.

**Table 3:** Blood NT-proBNP and the main hemodynamic parameters in decompensated cirrhosis depending on the presence of dilatation of the left ventricle and left atrium

	Left ventricular dilatation		p	Left atrial dilatation		p-value
	Present (n=14)	Absent (n=33)		Present (n=15)	Absent (n=32)	
NT-proBNP, pg/ml	115.3÷535.6	50.0÷196.6	0.042	196.6÷535.6	41.1÷168.9	*<* *0.001*
Ejection fraction, %	60.2±7.0	60.2±5.1	0.991	62.6±4.5	59.2±5.9	0.105
Stroke volume, ml	84.3±20.0	67.2±14.1	0.008	86.2±17.5	65.8±13.8	*<* *0.001*
Heart rate, bpm	87.2±10.7	83.2±11.1	0.301	87.9±11.5	82.8±10.8	0.189
Mean arterial pressure, mm Hg	81.4±8.9	86.1±6.1	0.085	82.0±5.9	86.0±7.6	0.085
Cardiac output, l/min	7.44±2.44	5.61±1.48	0.012	7.66±2.17	5.46±1.38	*0.001*
Systemic vascular resistance, dyn х sec/cm^5^	960±309	1319±383	0.004	933±309	1342±367	*0.001*
End-diastolic volume, ml	139.3±26.4	111.5±20.5	0.002	137.9±27.5	111.3±19.9	*0.003*
Hyperdynamic circulation (present/absent)	11/3	14/19	0.024	12/3	13/19	*0.012*

Significant differences are marked in italics.

Systolic heart dysfunction (ejection fraction < 50% (10)) was revealed in 2 patients but they had a NT-proBNP level in the blood of less than 125 (cut-off for heart failure (10)). It was interesting that the decrease in the ejection fraction was not accompanied by an increase in the level of NT-proBNP in the blood, as it takes place in heart failure. In patients with elevated blood NT-proBNP (>125 pg/ml (10)), end-diastolic volume, stroke volume, cardiac output, the volume of the left atrium were higher, systemic vascular resistance was lower, ejection fraction, blood pressure, and heart rate were not significantly differ, hyperdynamic circulation, dilatation left atrium, and diastolic dysfunction Grade II were more often revealed (Table 2). In patients with left ventricular dilatation (end-diastolic volume > 150 ml in men and > 106 ml in women (18)), the level of NT-proBNP was higher, and this was also accompanied by an increase in stroke volume, cardiac output and a decrease in systemic vascular resistance without significant differences in ejection fraction, heart rate and blood pressure. Patients with dilation of the left atrium (volume > 34 ml (18)) had similar changes (Table 3). Among 15 patients with dilation of the left atrium, 3 patients had diastolic dysfunction Grade I and 12 ones had diastolic dysfunction Grade II. 80% of patients with dilation of the left atrium had hyperdynamic circulation. The volume of the left atrium correlated with cardiac output (r = 0.471; p = 0.001).

**Figure 1:**
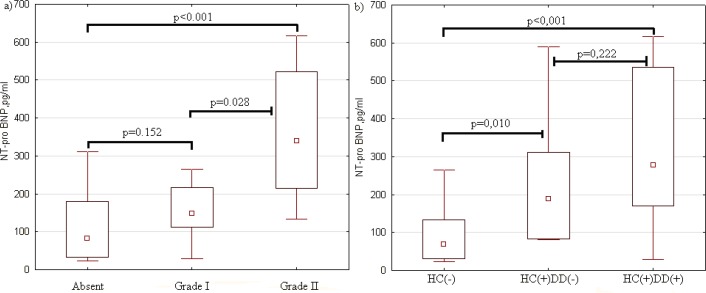
Blood NT-proBNP (pg / ml) in decompensated cirrhosis depending on the presence of diastolic dysfunction (a) and hyperdynamic circulation (b). HC(-) - patients without hyperdynamic circulation (n = 22), HC(+)DD(-) - patients with hyperdynamic circulation without diastolic dysfunction (n = 10), HC(+)DD (+) - patients with hyperdynamic circulation and diastolic dysfunction (n = 15). The point in the middle of each box represents the mean. The length of the box represents the mean±SD. The error bars show the non-outlier range

Diastolic dysfunction Grade I was not accompanied by a significant increase in blood NT-proBNP, in contrast to diastolic dysfunction Grade II (Figure 1a).

**Table 4:** Blood NT-proBNP and hemodynamic parameters in decompensated cirrhosis depending on the presence of hyperdynamic circulation

	Hyperdynamic circulation		p
	Present (n=25)	Absent (n=22)	
NT-proBNP, pg/ml	152÷476	31÷133	*<0.001*
End-diastolic volume, ml	134.5±23.7	103.0±15.5	*<0.001*
End-systolic volume, ml	50.4±11.2	44.2±11.0	0.072
Ejection fraction, %	62.6±3.6	57.6±7.4	*0.001*
Stroke volume, ml	84.1±14.9	58.9±8.7	*<0.001*
Heart rate, bpm	89.7±10.7	78.4±8.5	*0.001*
Systolic blood pressure, mm Hg	110.0±9.1	112±10.0	0.388
Diastolic blood pressure, mm Hg	70.8±7.0	72.5±9.6	0.440
Mean arterial pressure, mm Hg	83.9±7.2	85.7±7.5	0.337
Volume of the left atrium, ml	36.5±13.3	28.9±7.4	*0.043*
Left atrial dilatation (present/absent)	12/13	3/19	*0.012*
Systolic heart dysfunction	0	2	0.214
Diastolic heart dysfunction Grade I	5	3	0.427
Diastolic heart dysfunction Grade II	10	2	*0.016*
Systemic vascular resistance, dyn х sec/cm^5^	931±221	1532±301	*<0.001*

Significant differences are marked in italics

**Figure 2:**
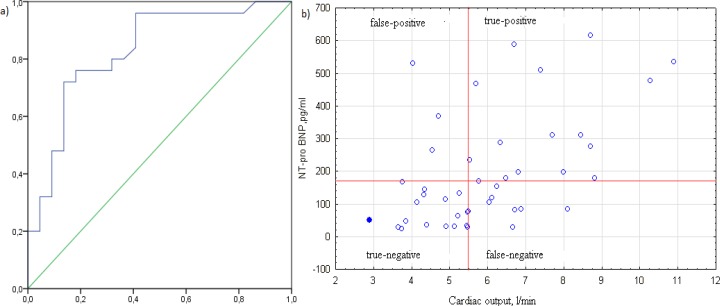
ROC analysis of blood NT-proBNP for detection of hyperdynamic circulation in decompensated cirrhosis (a) with a scattering diagram (b). Two truly positive results that significantly exceeded 700 pg/ml are not shown for the convenience of analyzing the scatter map

Based on our previous study, we determined that for our population, the upper value of the normal range of cardiac output is 5.5 l/min (16). Cardiac output above this level was regarded as evidence of hyperdynamic circulation, which was detected in 25 (53.2%) patients. In these patients, the level of NT-proBNP was higher, and this was also accompanied by an increase in end-diastolic volume, stroke volume, heart rate, left atrial volume and a decrease in systemic vascular resistance without significant difference in blood pressure. In patients with hyperdynamic circulation, dilatation of the left atrium and diastolic dysfunction Grade II were more often revealed. Interestingly, an increase in blood NT-proBNP in patients with hyperdynamic circulation was accompanied by an increase in ejection fraction, while in heart failure, opposite changes were described (20) (Table 4).

In patients with hyperdynamic circulation, blood NT-proBNP was higher, regardless of the presence of diastolic dysfunction, but in ones with diastolic dysfunction there was a tendency for higher NT-proBNP values (Figure 1b).

According to ROC analysis, the best cut-off values for detection of hyperdynamic circulation in decompensated cirrhosis are considered to be 170 pg/ml of blood NT-proBNP, showing sensitivity and specificity of 72.0 and 86.4%, respectively. The positive and negative predictive values were 86.4% and 73.1%. AUC = 0.829 (0.709-0.949), which correspond to a good biomarker characteristic (Figure 2).

## Discussion

An analysis of the results shows that an increase in blood NT-proBNP in decompensated cirrhosis is associated with an increase in the size of the left chambers of the heart as in heart failure. However, unlike the latter, in cirrhosis an increase in blood NT-proBNP are accompanied by an increase in stroke volume and cardiac output and a decrease in systemic vascular resistance without a significant change in ejection fraction. In decompensated cirrhosis, blood NT-proBNP directly correlates with end-diastolic volume, stroke volume, cardiac output, volume of the left atrium, inversely correlates with systemic vascular resistance, and does not correlate with ejection fraction, whereas in heart failure, a negative correlation with ejection fraction is shown (20). In contrast, patients with decompensated cirrhosis have a tendency to increase ejection fraction with an increase in blood NT-proBNP.

The findings allow suggesting that the pathogenesis of the increase in blood NT-proBNP in decompensated cirrhosis and systolic heart failure is different, but in both cases is associated with stretching of left ventricular cardiomyocytes.

In systolic heart failure, ejection fraction decreases which leads to decrease in stroke volume and, consequently, cardiac output. As a compensatory reaction in accordance with Starling's law, end-diastolic volume increases to increase stroke volume and cardiac output, but this is not always sufficient to normalize these. Therefore, an increase in NT-proBNP in systolic cardiac dysfunction is accompanied by a decrease in stroke volume and cardiac output and a compensatory increase in systemic vascular resistance to maintain blood pressure. In decompensated cirrhosis, reverse changes are observed. Endotoxemia leads to arterial vasodilation which leads to a decrease in systemic vascular resistance and blood pressure (21). In response to a decrease in blood pressure, barostatic mechanisms are activated, leading to fluid retention, which leads to increase in venous return and end-diastolic volume. These lead to an increase in stroke volume and cardiac output. Therefore, hyperdynamic circulation develops.

Thus, we showed that blood NT-proBNP in decompensated cirrhosis is a biomarker for hyperdynamic circulation, but not a biomarker for systolic cardiac dysfunction. As a result of the ROC analysis, we proposed a cut-off point for the value of this biomarker (170.0 pg/ml), which allows to identify patients with hyperdynamic circulation with high analytical reliability.

In one of the previous studies (12), there was no correlation between NT-proBNP and cardiac output in cirrhosis. The contradiction with our findings can be explained by the fact that in the cited work the authors did not divide patients into groups with compensated and decompensated cirrhosis, while hyperdynamic circulation was observed almost exclusively in patients with decompensated cirrhosis (16). In addition, if we analyze the regression curve of cardiac output to NT-proBNP, it can be seen that the correlation between these begins with a cardiac output level > 4.0 l/min, whereas cardiac output does not exceed this level in 42% of patients with the compensated cirrhosis (16).

In another study (13), it was shown that an increase in NT-proBNP is associated with diastolic dysfunction in cirrhosis. In our study, blood NT-proBNP was higher only in diastolic dysfunction Grade II when the left atrium volume increases, but not in diastolic dysfunction Grade I when, in most cases (62.5% in our work), dilation of left atrium is not observed. We suggest these due to the fact that blood NT-proBNP increases also as result of the extension of cardiomyocytes of left atrium, but not only as the result of the extension of cardiomyocytes of left ventricle. But the muscle mass of the left atrium less than muscle mass of the left ventricle. Therefore, the myocardium of the left atrium produces less NT-proBNP than myocardium of the left ventricle. At the same time, according to our findings, the increase in blood NT-proBNP in hyperdynamic circulation does not depend on the presence of diastolic dysfunction, but in patients with hyperdynamic circulation and diastolic dysfunction there is a tendency to higher blood NT-proBNP than in patients with hyperdynamic circulation but without diastolic dysfunction.

The use of NT-proBNP as a simple serum biomarker for hyperdynamic circulation may provide differential management with beta-blockers and control for their use, which may increase the effectiveness of therapy and reduce the incidence of side effects. Further searches are required to test this hypothesis.

## Conflict of interests

The authors declare that they have no conflict of interest.
